# Pulmonary Thrombosis or Embolism in a Large Cohort of Hospitalized Patients With Covid-19

**DOI:** 10.3389/fmed.2020.00557

**Published:** 2020-08-25

**Authors:** Natividad Benito, David Filella, Jose Mateo, Ana M. Fortuna, Juan E. Gutierrez-Alliende, Nerea Hernandez, Ana M. Gimenez, Virginia Pomar, Ivan Castellvi, Hector Corominas, Jordi Casademont, Pere Domingo

**Affiliations:** ^1^Infectious Disease Unit, Hospital de la Santa Creu i Sant Pau - Institut d'Investigació Biomèdica Sant Pau, Barcelona, Spain; ^2^Department of Medicine, Universitat Autònoma de Barcelona, Barcelona, Spain; ^3^Department of Internal Medicine, Hospital de la Santa Creu i Sant Pau - Institut d'Investigació Biomèdica Sant Pau, Barcelona, Spain; ^4^Thrombosis and Hemostasis Unit, Hospital de la Santa Creu i Sant Pau - Institut d'Investigació Biomèdica Sant Pau, Barcelona, Spain; ^5^Department of Respiratory Diseases, Hospital de la Santa Creu i Sant Pau - Institut d'Investigació Biomèdica Sant Pau, Barcelona, Spain; ^6^Department of Radiology, Hospital de la Santa Creu i Sant Pau - Institut d'Investigació Biomèdica Sant Pau, Barcelona, Spain; ^7^Department of Rheumatology, Hospital de la Santa Creu i Sant Pau - Institut d'Investigació Biomèdica Sant Pau, Barcelona, Spain

**Keywords:** COVID-19, pulmonary thrombosis, pulmonary embolism (MeSH), thromboprophylaxis, anticoagulant (MeSH), thromboinflammation

## Abstract

**Objective:** We set out to analyze the incidence and predictive factors of pulmonary embolism (PE) in hospitalized patients with Covid-19.

**Methods:** We prospectively collected data from all consecutive patients with laboratory-confirmed Covid-19 admitted to the Hospital de la Santa Creu i Sant Pau, a university hospital in Barcelona, between March 9 and April 15, 2020. Patients with suspected PE, according to standardized guidelines, underwent CT pulmonary angiography (CTPA).

**Results:** A total of 1,275 patients with Covid-19 were admitted to hospital. CTPA was performed on 76 inpatients, and a diagnosis of PE was made in 32 (2.6% [95%CI 1.7–3.5%]). Patients with PE were older, and they exhibited lower PaO_2_:FiO_2_ ratios and higher levels of D-dimer and C-reactive protein (CRP). They more often required admission to ICU and mechanical ventilation, and they often had longer hospital stays, although in-hospital mortality was no greater than in patients without PE. High CRP and D-dimer levels at admission (≥150 mg/L and ≥1,000 ng/ml, respectively) and a peak D-dimer ≥6,000 ng/ml during hospital stay were independent factors associated with PE. Prophylactic low molecular weight heparin did not appear to prevent PE. Increased CRP levels correlated with increased D-dimer levels and both correlated with a lower PaO_2_:FiO_2_.

**Conclusions:** The 2.6% incidence of PE in Covid-19 hospitalized patients is clearly high. Higher doses of thromboprophylaxis may be required to prevent PE, particularly in patients at increased risk, such as those with high levels of CRP and D-dimer at admission. These findings should be validated in future studies.

## Introduction

Since December 2019, the rapid spread of the novel betacoronavirus, named SARS-CoV-2, has led to a global pandemic of coronavirus disease 2019 (Covid-19). Several recent studies have shown that patients with Covid-19 frequently have coagulation disorders, especially a marked increase in D-dimer (1–5). These abnormal coagulation parameters have been associated with worse outcomes (1, 2). It is also suggested that Covid-19 predisposes patients to a higher risk of thrombotic disorders, including both venous and arterial thromboembolic disease (3, 4). Several scientific societies and authors have already proposed specific guidelines and recommendations on the use of thromboprophylaxis in patients with Covid-19 (6–11), although the best thromboprophylaxis regimen (dosing and duration), taking into account the characteristics of different groups of patients, has not been established.

To date, a few case series studies (12–19) and some case reports (17, 20–30) have investigated the incidence and features of pulmonary thrombosis or embolism (PE) in patients with Covid-19. All studies included small numbers of cases, and most of them were conducted in ICU patients (12, 16–19), as they constitute a particular group with an increased risk of PE (14). Other studies have been based on patients who underwent computed tomography pulmonary arteriography (CTPA) but did not provide clear information on the baseline population (14, 15). These limitations make it difficult to know the true incidence, characteristics, and risk factors of PE in patients with Covid-19.

Using a large cohort of consecutive patients with laboratory-confirmed Covid-19 admitted to a single university hospital, we sought to analyze the incidence, clinical features, and predictive factors of PE in patients with Covid-19.

## Methods

### Setting

We conducted this study at the Hospital de la Santa Creu i Sant Pau, a tertiary acute care university hospital in Barcelona, Spain. The hospital's Institutional Review Board approved the study.

We collected data prospectively from all consecutive patients with laboratory-confirmed Covid-19 diagnosed at our hospital between March 9 and April 15, 2020, and we included those who were admitted to hospital.

Specific guidance based on the International Society of Thrombosis and Hemostasis interim guidelines (6) was developed at our center and distributed on March 18 to all attending staff. It was recommended to start prophylactic doses of subcutaneous low molecular weight heparin (LMWH) (enoxaparin 4,000 IU/24 h, bemiparin 3,500 IU/24 h or tinzaparin 4,500 IU/24 h) for all patients (including the non-critically ill) who required hospital admission for Covid-19 in the absence of contraindications. Doses were adjusted to body weight. On April 3, 2020, the guidance was subsequently amplified by adding the use of high-dose prophylaxis for patients at increased thrombotic risk, including those with D-dimer >3,000 ng/mL (enoxaparin 100 IU/kg/ 24 h, bemiparin 75–80 IU/kg/24 h, or tinzaparin 100 IU/Kg/ 24 h).

Hospital guidance on when to suspect possible PE and request a CTPA in Covid-19 patients was also developed and distributed to all attending staff. Suspicion of having PE included those patients whose partial pressure of arterial oxygen to fraction of inspired oxygen (PaO_2_:FiO_2_) ratio worsened or failed to improve, associated with an increasing or persistently high D-dimer (>3,000 ng/mL) and/or hemodynamic deterioration or other “classic” symptoms of PE, such as pleuritic chest pain, hemoptysis, syncope, and/or signs of right ventricular strain. Patients with suspicion of PE had a CTPA and started anticoagulant therapy with full-dose LMWH if PE was diagnosed.

All CTPA scans were performed using a 16-slice multi-detector CT (Philips Brilliance CT 16 C Slice) after intravenous injection of 60 ml iodinated contrast agent (Optiray Ultraject 350 mg/mL Ioversol, Guerbet, France) at a flow rate of 4 mL/s, triggered on the main pulmonary artery. The CT scan settings were 120 kVp, slice thickness 2 mm, increment 1 mm, pitch 0.688, rotation time 0.28 s, and average tube current 300 mA. The location of embolus (main pulmonary, lobar, segmental, and subsegmental artery) and clot burden (low, moderate, and high) according to a modified Qanadli Score (31) were evaluated. Right ventricle overload was also assessed (right ventricle diameter to left ventricle diameter ratio >1.3). Following the clinical guidelines developed at our medical center and the recommendations of the Spanish Agency for Medicinal Products and Medical Devices (AEMPS), a single dose of intravenous tocilizumab (600 mg for patients ≥75 kg; 400 mg for those <75 kg) was suggested as treatment for hospitalized patients with Covid-19 and data for cytokine release syndrome.

### Variables Assessed

We collected information on patients who underwent CTPA for suspected PE. Variables included demographic data, preexisting chronic medical conditions, body mass index, thrombosis prophylaxis with LMWH and dosing, diagnosis and characteristics of PE, PaO_2_:FiO_2_ ratio, estimated using methods developed by Brown and colleagues (32, 33) (PaO_2_:FiO_2_ at the time of CTPA and worst ratio during hospital stay), evolution of D-dimer and C-reactive protein (at admission and peak level), and clinical outcomes, including need for invasive mechanical ventilation, ICU admission, length of hospital stay, and in-hospital death.

### Statistical Analysis

We summarized continuous variables as medians and interquartile ranges and categorical variables as percentages of the total sample for that variable. The incidence of PE was estimated with a 95% confidence interval (CI). We used the Wilcoxon rank-sum and chi-square tests (or Fisher's exact tests when appropriate) to evaluate group differences (Covid-19 patients with and without PE in the CTPA) for continuous and categorical variables, respectively. A multivariable logistic regression model was used to identify factors independently associated with a higher risk of developing PE. Any variable tested in univariate analysis with a *p* < 0.25, together with all variables of known clinical importance, were selected as candidates for the first multivariate model. We then followed the purposeful selection of covariates method described by Hosmer et al. (34) Final parameter estimates are shown as odds ratios (OR) with their corresponding 95% CIs. Correlations between quantitative variables were examined using the Spearman rank correlation test. *P* < 0.05 were considered to be significant for all statistical tests. Data were analyzed using IBM® SPSS®, version 26.0.

## Results

Of 1,863 consecutive patients diagnosed with laboratory-confirmed Covid-19 between March 9 and April 15, 2020, at our center, a total of 1,275 patients (68.4%) were admitted to hospital. A total of 146 inpatients (12.4%; 95% CI 10.5–14.4%) died during their hospital stay [101 patients (7.9%) were still hospitalized at the time of data analysis].

During this period, a CTPA was indicated for suspicion of PE in 76 inpatients (6% of patients with Covid-19 admitted to hospital). CTPA confirmed a diagnosis of PE in 32 patients (42.1% of tests), which represents a cumulative incidence of 2.6% (95 CI 1.7–3.5%) for PE among Covid-19 inpatients. Most CTPAs were requested for patients admitted to conventional wards (70%); in fact, a similar percentage of patients with (71.9%) and without PE (68.2%) were hospitalized in the wards at the time of diagnosis (Table 1).

**Table 1:** Characteristics of patients with Covid-19 with and without pulmonary embolism.

**Variable**	**Pulmonary embolism *N* = 32**	**NO pulmonary embolism *N* = 44**	***P*-value**
Age, years—median (IQR)	66 (13)	60 (17)	0.110
Age ≥ 60 years—no. (%)	24 (75)	22 (50)	**0.028**
Male gender—no. (%)	20 (62.5)	31 (70.5)	0.466
Diabetes mellitus—no. (%)	6 (18.8)	6 (13.6)	0.546
Hypertension—no. (%)	15 (46.9)	19 (43.2)	0.749
Chronic lung disease—no. (%)	4 (12.5)	12 (27.3)	0.119
Active cancer—no. (%)	5 (15.6)	2 (4.5)	0.124
BMI—median (IQR)	28.1 (5.4)	26.7 (8)	0.527
BMI ≥ 25—no. (%)	22 (75.9)	27 (71.1)	0.660
Obesity—no. (%)	8 (27.6)	13 (32.5)	0.661
Area of admission in the hospital at the time of performing CTPA—no. (%)			0.938
- emergency department - inpatient ward - intensive care unit	4 (12.5) 23 (71.9) 5 (15.6)	6 (13.6) 30 (68.2) 8 (18.2)	
Days from admission until CTPA performed—median (IQR)	7 (10.5)	5 (10)	0.398
LMWH administration before CTPA—no. (%)	28 (87.5)	39 (88.6)	1
Days of LMWH administration before CTPA—median (IQR)	6 (9)	5 (9)	0.922
LMWH doses—no. (%)			0.823
- prophylactic doses - “higher risk” prophylactic doses - therapeutic doses	26 (92.9) 2 (7.1) 0 (0)	34 (87.2) 4 (10.3) 1 (2.6)	
Pneumonia in the CTPA—no. (%)	31 (96.9)	41 (93.2)	0.634
PaO_2_:FiO_2_ at the time of performing CTPA	222 (163.5)	250 (181.8)	0.102
Invasive mechanical ventilation at the time of CTPA—no. (%)	7 (21.9)	6 (13.6)	0.346
Worst PaO_2_:FiO_2_–median (IQR)	158.5 (134.8)	228.5 (170.5)	**0.017**
Patients needing ICU admission during their hospital stay—no. (%)	15 (46.9)	10 (22.7)	**0.027**
Patients requiring invasive mechanical ventilation during their hospital stay—no. (%)	14 (43.8)	8 (18.2)	**0.015**
D-dimer at admission, ng/mL—median (IQR)	5,274.5 (16,419)	1,045.5 (6,287.8)	**0.017**
D-dimer at admission ≥ 1,000 ng/mL—no. (%)	26 (81.3)	23 (52.3)	**0.009**
Peak D-dimer, ng/mL—median (IQR)	22,791 (42,552)	6039.5 (15,982.3)	**0.001**
Peak D-dimer ≥ 3,000 ng/mL—no. (%)	31 (96.9)	29 (65.9)	**0.001**
Peak D-dimer ≥ 6,000 ng/mL—no. (%)	29 (90.6)	23 (52.3)	**<0.001**
C-reactive protein at admission, mg/L—median (IQR)	161.5 (65)	83.5 (126)	**0.007**
C-reactive protein at admission ≥ 150 mg/L—no. (%)	24 (75)	14 (31.8)	**<0.001**
Peak C-reactive protein, mg/L—median (IQR)	221.5 (169.4)	183 (236.4)	0.059
Hospital stay, days—median (IQR)	15.5 (9.8)	7 (11)	**0.010**
In-hospital death—no. (%)	3 (9.4)	5 (11.4)	1

*BMI, body mass index; CTPA, computed tomography pulmonary arteriography; FiO_2_, Fraction of inspired oxygen; IQR, interquartile range; LMWH, low molecular weight heparin; PaO_2_ partial pressure of arterial oxygen*.

*Bold values refer to P-values that are statistically significant (<0.05); the idea is to make easer the identification of statistically significant variables in this large table*.

Table 1 shows the characteristics of Covid-19 inpatients with and without PE in the CTPA. Patients with PE were more often ≥60 years old, had lower PaO_2_:FiO_2_ ratios, and had higher levels of D-dimer and C-reactive protein. The D-dimer levels of these patients were therefore more than five times higher at hospital admission, and the peak D-dimer level during hospital stay was more than three times higher than in patients without PE. Moreover, C-reactive protein at admission was almost twice as high among PE patients. Covid-19 inpatients with PE more often required ICU admission and invasive mechanical ventilation than those without PE and had longer hospital stays, although in-hospital mortality was not statistically significantly different between the two groups (9.4% vs. 11.4%). There were also no significant differences between in-hospital mortality in PE patients and mortality in all admitted Covid-19 patients (*p* = 0.604). A high proportion of patients with and without PE (87.5% and 88.6, respectively) were receiving LMWH prior to CTPA, mostly at prophylactic doses, with no statistically significant differences between the two groups.

We did not investigate deep vein thrombosis (DVT), except in symptomatic patients. We found DVT in two patients with PE, one of whom was a patient with gastric cancer.

Candidate variables for the first multivariable model were: age ≥ 60 years, sex, chronic lung disease, active cancer, BMI, LMWH administration, D-dimer at admission ≥ 1,000 ng/mL, C-reactive protein at admission ≥ 150 mg/L, ICU admission, worst PaO_2_:FiO_2_, and peak D-dimer ≥ 6,000. Multivariate analysis found the following independent factors as associated with an increased risk of having PE: C-reactive protein at admission ≥ 150 mg/L (OR 7.9, 95% CI 2.4–26.7), D-dimer at admission ≥ 1,000 ng/mL (OR 4.5, 95% CI 1.2–17.2, *p* = 0.026), and peak D-dimer ≥ 6,000 ng/mL during hospital stay (OR 5.6, 95% CI 1.3–24.5) (Nagelkerke *R*^2^ = 0.439).

**Figure 1:**
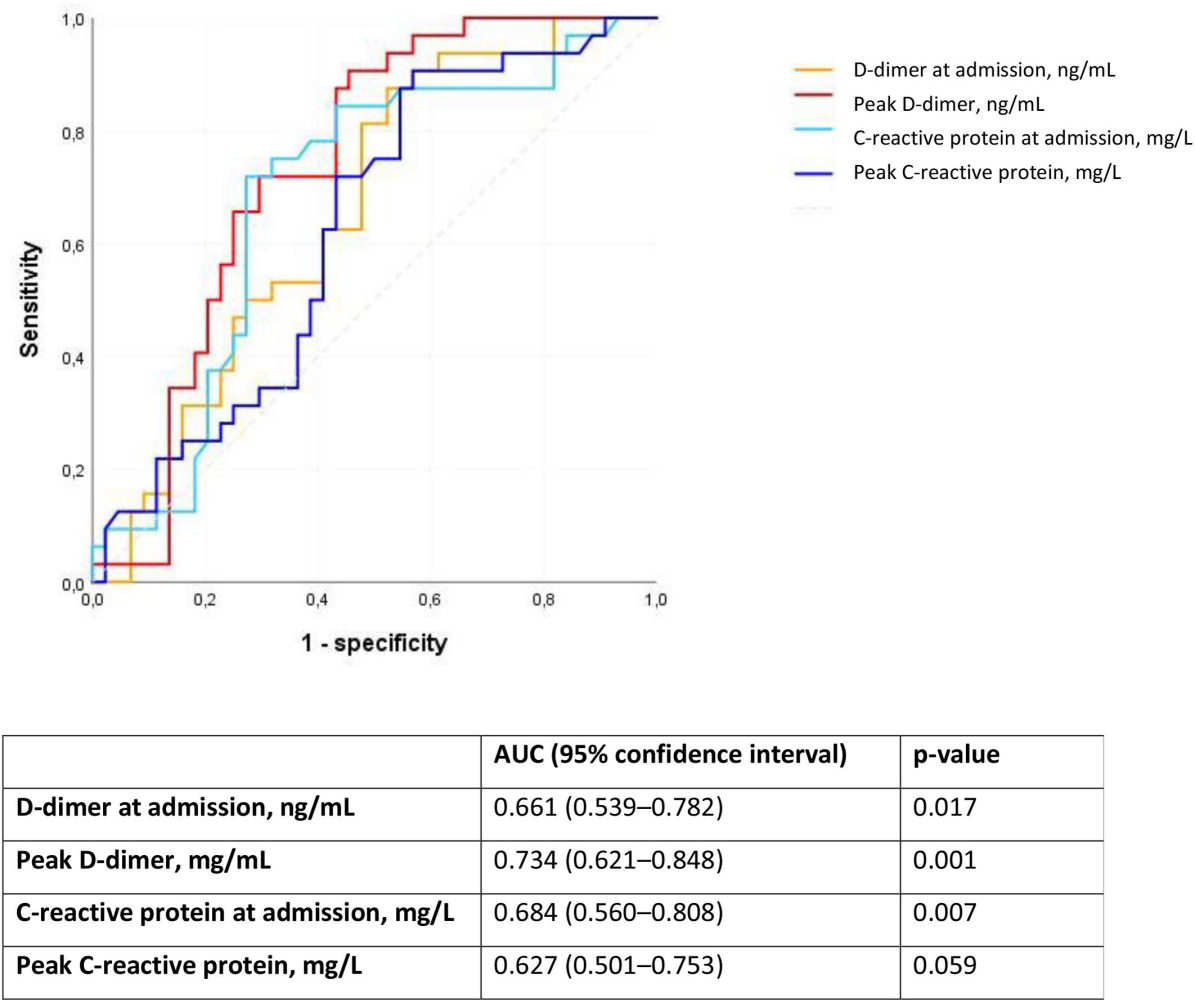
Receiver operating characteristic (ROC) curve for D-dimer and C-reactive protein as predictors of pulmonary embolism in patients with Covid-19. AUC, area under the ROC curve.

We analyzed D-dimer and C-reactive protein as predictors of PE using ROC curves. The peak D-dimer during hospital stay followed by C-reactive protein at admission had the largest area under the ROC curve (Figure 1).

**Figure 2:**
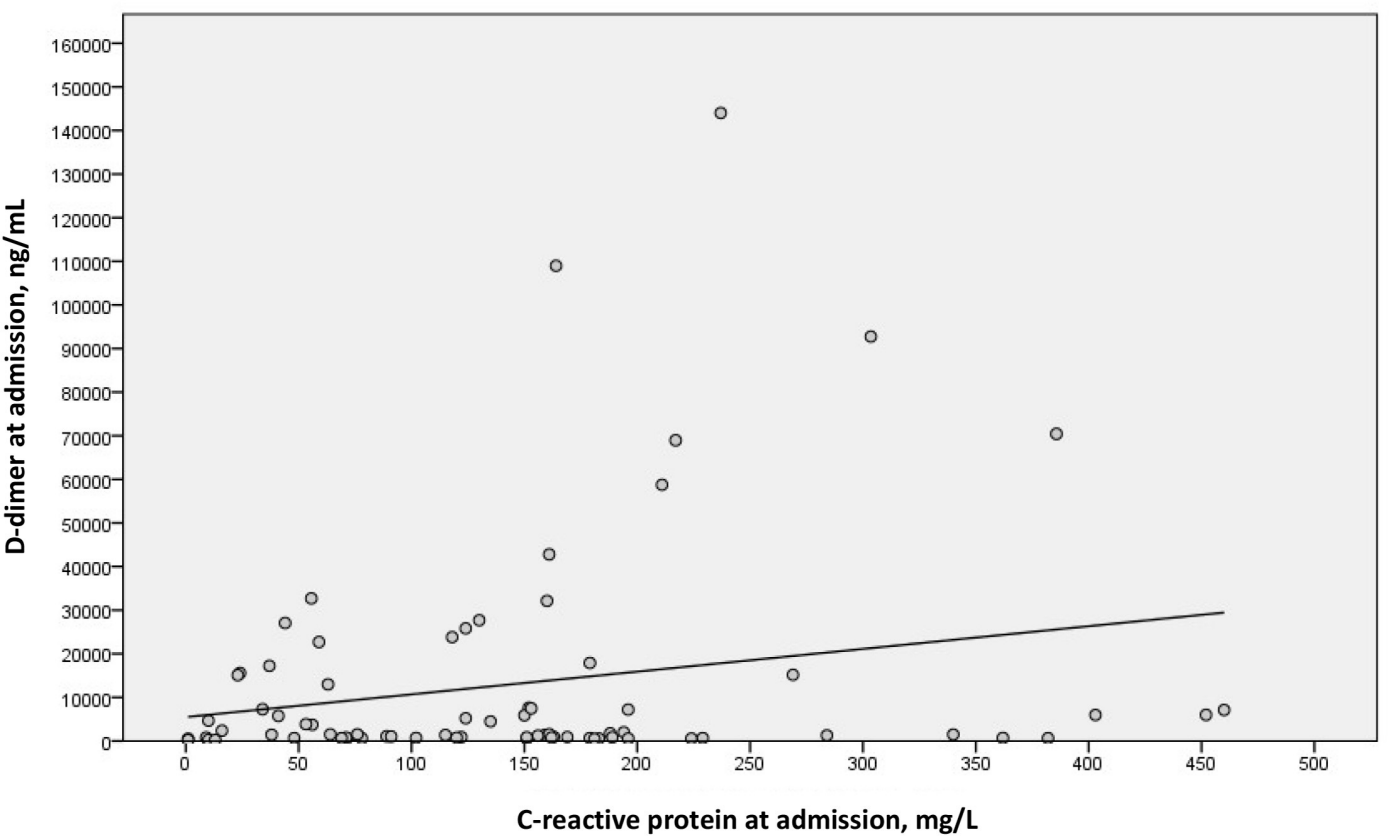
Correlation between D-dimer and C-reactive protein at admission in patients with Covid-19 and suspected pulmonary embolism (r_s_ = 0.146; *p* = 0.208).

**Figure 3:**
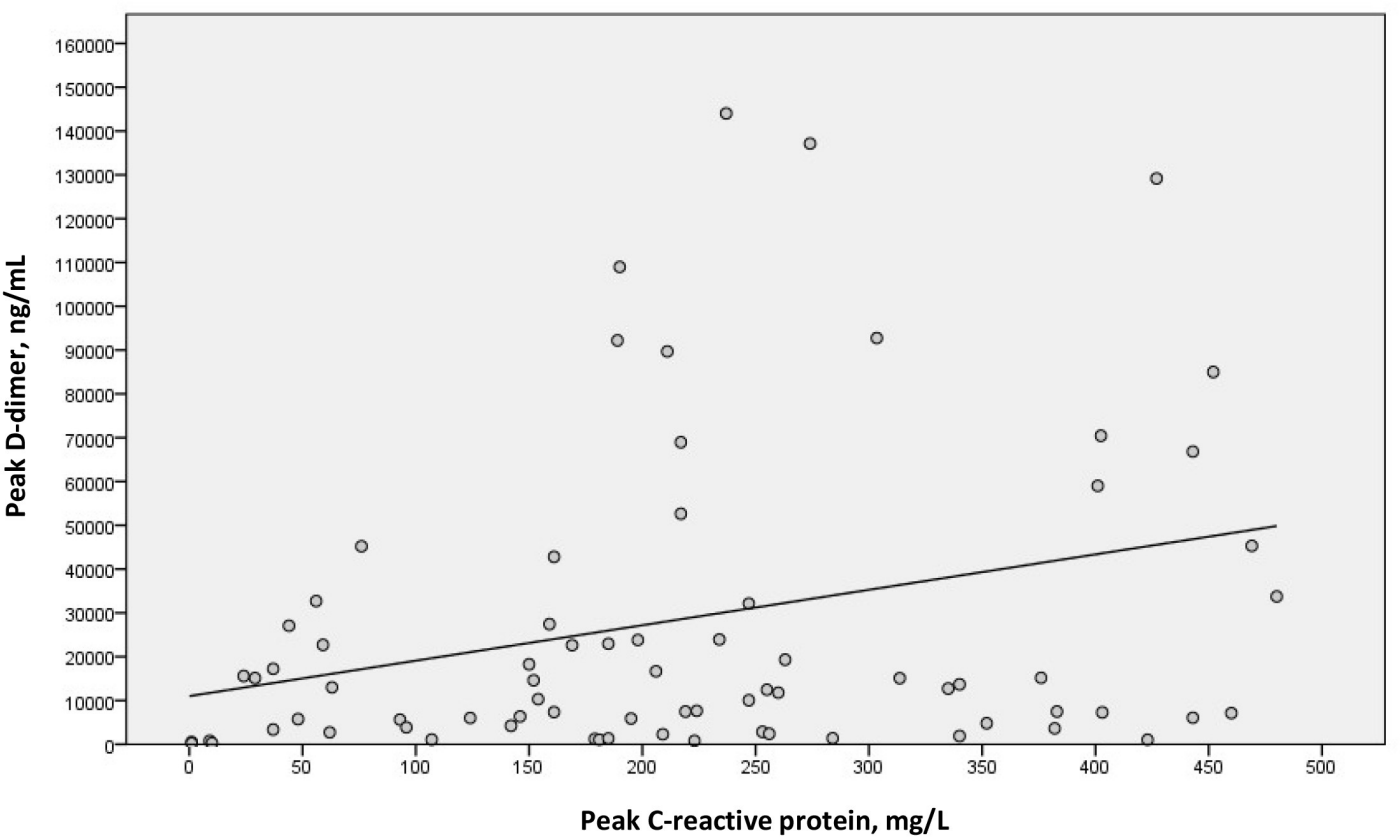
Correlation between peak D-dimer and peak C-reactive protein during hospital stay in patients with Covid-19 and suspected pulmonary embolism (r_s_ = 0.279; *p* = 0.015).

**Figure 4:**
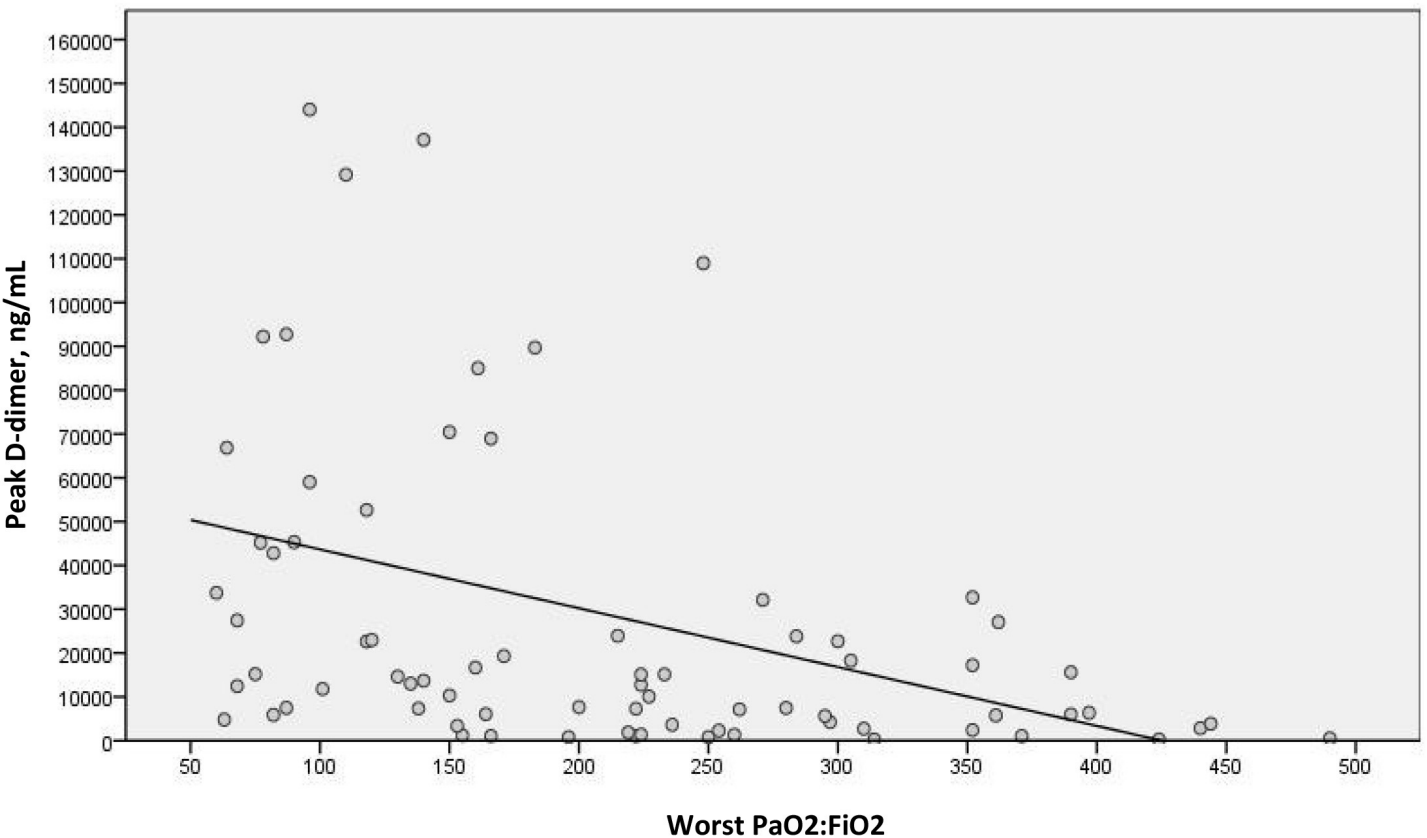
Correlation between peak D-dimer and worst PaO_2_:FiO_2_ during hospital stay in patients with COVID-19 and suspected pulmonary embolism (r_s_ = −0.471; *p* < 0.001).

**Figure 5:**
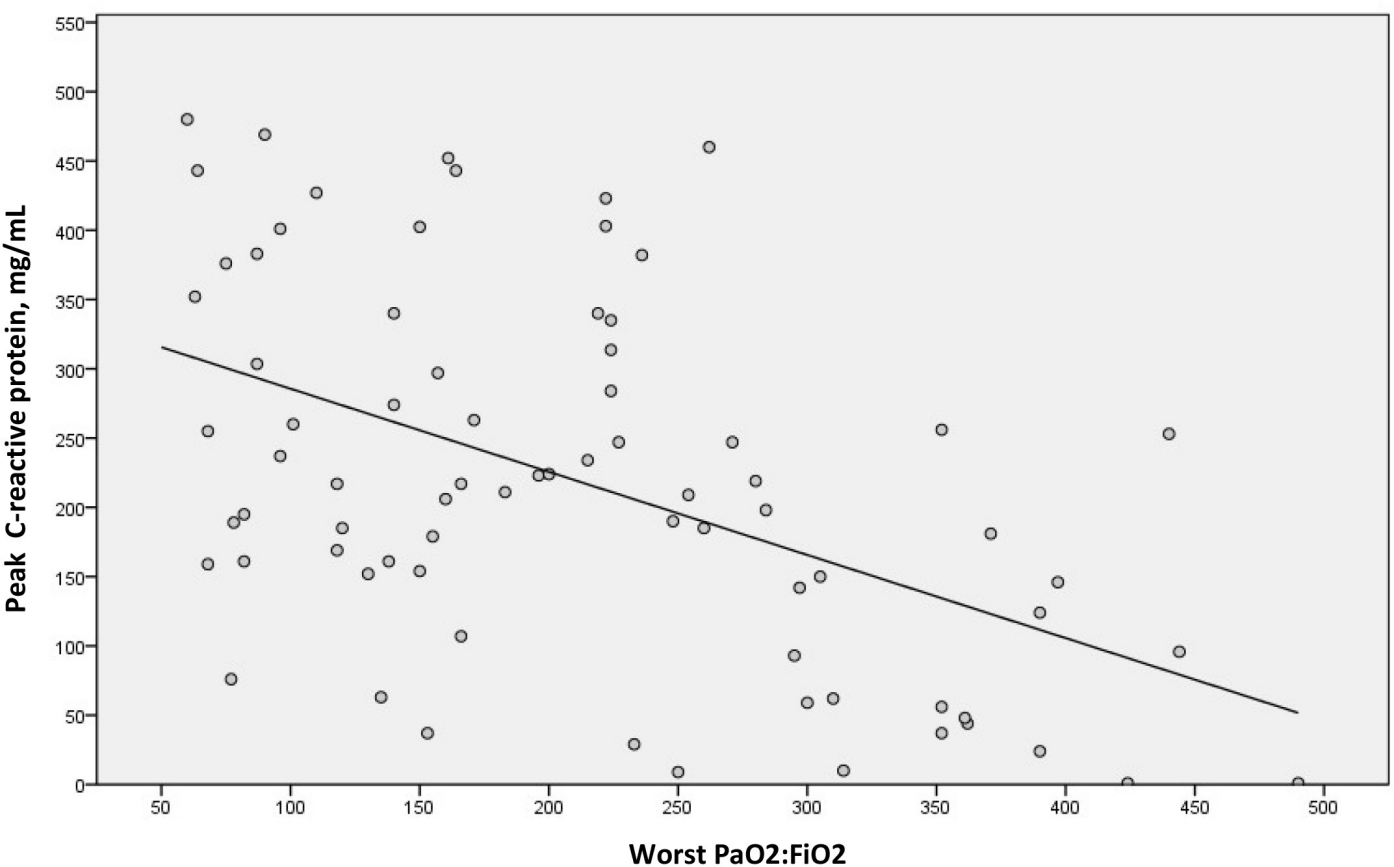
Correlation between highest C-reactive protein and worst PaO_2_:FiO_2_ during hospital stay in patients with COVID-19 and suspected pulmonary embolism (r_s_ = −0.473; *p* < 0.001).

We examined a possible linear correlation between levels of D-dimer and C-reactive protein at admission (Figure 2) and at their highest levels during hospital stay (Figure 3). A positive linear correlation was observed in both cases and was statistically significant for peak D-dimer and peak C-reactive protein values during hospitalization. We also found statistically significant negative correlations between peak D-dimer levels and worst PaO_2_:FiO_2_ ratio (Figure 4) and between peak C-reactive protein levels and worst PaO_2_:FiO_2_ ratio (Figure 5) during hospital stay.

In patients with PE, the CTPA showed mainly affection of the segmental and subsegmental branches of pulmonary arteries, predominantly with a low thrombus load (Table 2).

**Table 2:** Characteristics of pulmonary embolism in the computed tomography pulmonary angiogram of patients with Covid-19.

**Variable**	**Patients with pulmonary embolism (*n* = 32)**
**Location of embolus - no. (%)**	
- Main pulmonary artery - Lobar artery - Segmental artery - Subsegmental artery	0 7 (21.9) 16 (50) 9 (28.1)
**Thrombus load - no. (%)**	
- High - Moderate - Low	4 (12.5) 8 (25) 20 (62.5)
**Right ventricular overload - no. (%)**	5 (15.6)

## Discussion

In this analysis involving a large sample of consecutive patients hospitalized with Covid-19, we found a high incidence of PE of 2.6% despite the wide use of thromboprophylaxis. Patients with PE were older and had lower PaO_2_:FiO_2_ ratios and markedly higher levels of D-dimer and C-reactive protein. They more often required admission to ICU, invasive mechanical ventilation, and longer hospital stays, although in-hospital mortality was no greater than in patients without PE. We identified high C-reactive protein and D-dimer at admission (≥150 mg/L and ≥1,000 ng/ml, respectively) and a peak D-dimer during hospital stay ≥6,000 ng/ml as independent factors associated with PE. Prophylactic doses of LMWH did not, however, appear to prevent PE.

Most of the studies on PE in patients with Covid-19 have been conducted in ICU patients, who are at greater risk of venous thromboembolism and have shown incidences ranging from 17 to 35% (12, 16–19, 35). Interestingly, one of these studies demonstrated that patients with acute respiratory distress syndrome (ARDS) due to Covid-19 developed PE significantly more often than patients with ARDS due to other diseases (11.7% vs. 2.1%) (18). Another study showed a much higher frequency of PE in ICU patients with Covid-19 (21%) than during the same time interval in 2019 (6%), and it was also higher than the incidence of PE in patients with influenza admitted to the same ICU in 2019 (8%) (12). It seems clear therefore that the incidence of PE in patients admitted to ICU with Covid-19 is much higher than in other critically ill non-Covid-19 patients, including those with ARDS and other respiratory infections, despite the fact that these patients are already at an increased risk of PE (10).

The only previous study that has addressed the incidence of PE in all patients admitted to hospital with Covid-19 found a percentage of 2.8% (13). Despite the smaller sample size, this proportion is remarkably similar to that found in the present study (2.6%). In both studies, the vast majority of patients (>70%) were admitted to conventional wards. An incidence of PE close to 3% (which may be underestimated by the number of patients undergoing CTPA) is certainly high, particularly when compared with the figure of 1.4% for PE found in a large prospective study of critically ill patients in the ICU (36).

In most of the studies mentioned above, as was the case in the present study, patients developed PE even though most of them were receiving anticoagulant thromboprophylaxis. These findings raise the need to increase thromboprophylaxis doses, particularly in higher risk patients (5, 16, 18). The predictive factors for PE found in the present study can help identify patients who may benefit from high prophylactic doses. Patients with C-reactive protein ≥ 150 mg/L and D-dimer ≥ 1,000 could therefore be candidates for increased doses of thromboprophylaxis; if these patients continue to have a persistently elevated or increasing D-dimer, however, CTPA should be considered to diagnose possible PE.

When analyzing all patients with suspicion of PE, we found that increased C-reactive protein levels (an acute phase protein whose serum concentrations increase during inflammatory states and whose expression is driven by IL-6) (37) correlated with increased D-dimer levels, suggesting a link between inflammation and procoagulant changes, as proposed by other authors (5, 38). In fact, increased C-reactive protein (≥150 mg/mL) at admission was the strongest predictor of developing PE in multivariable analysis, although peak D-dimer had greater diagnostic capacity according to the ROC curve. This supports the hypothesis that inflammation associated with Covid-19 leads to subsequent activation of coagulation and a higher risk of thrombotic disease (4, 38). Furthermore, we also demonstrated that both high levels of D-dimer and C-reactive protein correlated with increased hypoxemia, evidenced by a decrease in the PaO_2_:FiO_2_ ratio. This provides further important information about the hypercoagulability and thromboinflammatory response associated with Covid-19 and their association with acute lung injury.

A relevant debate has arisen recently about whether thrombosis or pulmonary embolism is the most critical aspect of pulmonary thromboembolic events in Covid-19 patients (16, 39, 40). These patients are clearly at increased risk for venous thromboembolic disease (41), and it is therefore not surprising that they may also develop pulmonary embolisms. Pulmonary thrombosis in Covid-19 patients found at autopsy (42) and, remarkably, in all studied patients in one very recent study (40), however, suggests that pulmonary thrombosis may play a role in the pathogenesis of more severe cases. Our study does not permit to differentiate between thrombosis and pulmonary embolism. Nevertheless, some of our findings would support the predominance of pulmonary thrombosis. First, the low frequency of patients with DVT is worthy of note, which is consistent with other studies (12, 16). Likewise, the predominant involvement in our study of the segmental and subsegmental pulmonary arteries, together with the association between inflammation, coagulation, and hypoxemia, would also support this hypothesis.

Our study has limitations that are mainly associated with its observational study design. With such a large number of patients and the high demand for care during this period, systematic performance of CTPA in all patients was not possible, which may have led to underestimating the true incidence of PE. Either way, any large prospective study including all Covid-19 hospitalized patients should probably be based on establishing criteria by which to evaluate CTPA requests since it does not seem feasible for all patients to have a CTPA. To enable the standardization of criteria, our institution developed hospital guidelines on when to suspect PE and request a CTPA. On the other hand, we compared the features of patients with and without PE on the basis of their CTPA results so that our findings cannot be extrapolated to the entire cohort of inpatients with Covid-19. In order to obtain results that apply to all hospitalized patients with Covid-19, we are currently planning a nested case-cohort study that will include all PE patients diagnosed by CTPA as cases and a random sample of all Covid-19 inpatients as controls. The CT unit used in our study was a Phillips Brilliance CT 16-slice scanner, which is the one reserved at our center for emergencies. During the Covid-19 pandemic, this CT unit was reserved for the study of all patients with Covid-19 in order to spare the other CT units. While better CT image resolution would be obtained with other more technologically up-to-date units, we think that the quality of the image, even in subsegmental arteries, is sufficient for cases of PE. Even so, while the frequency of PE found is high, it is probably underestimated, as the data from autopsies of patients with Covid-19 suggest (40, 43). In an autopsy study of 12 consecutive patients who died from Covid-19, PE was found in five of them even though no preclinical evidence of PE had been reported (43). In another study, meticulous autopsies of 11 deceased patients (10 of whom were selected at random) observed thrombosis in small to mid-sized pulmonary arteries in all cases (40).

In conclusion, patients admitted to hospital with Covid-19 have a high incidence of PE, estimated at 2.6%. Our study identified predictors of PE able to select patients at increased risk of developing PE, making them possible candidates for thromboprophylaxis at higher doses. There is a correlation between increased levels of C-reactive protein and D-dimer and increased hypoxemia, which supports the role of thromboinflammation in acute lung injury observed in patients with Covid-19. We need more information on the most appropriate thromboprophylactic doses and duration to prevent PE in patients with Covid-19.

## Data Availability Statement

The data that support the findings of this study are available from the corresponding author upon reasonable request.

## Ethics Statement

The studies involving human participants were reviewed and approved by Clinical Research Ethics Committee of the Hospital de la Santa Creu i Sant Pau. Written informed consent for participation was not required for this study in accordance with the national legislation and the institutional requirements.

## Author Contributions

NB, DF, and JM conceived the study, searched the literature, and designed the study. NB analyzed the data and drafted the report. NB, DF, JM, and AF interpreted the results. DF, VP, JG-A, and AG collected the data and critically revised the report for important intellectual content. DF, JM, and AF critically revised the report. JC and PD supervised the study, interpreted the results, and critically revised the report for important intellectual content. All authors gave final approval of the version to be published.

## Conflict of Interest

The authors declare that the research was conducted in the absence of any commercial or financial relationships that could be construed as a potential conflict of interest.
